# Ticks and serosurvey of anti-*Rickettsia* spp. antibodies in wild boars (*Sus scrofa*), hunting dogs and hunters of Brazil

**DOI:** 10.1371/journal.pntd.0007405

**Published:** 2019-05-30

**Authors:** Louise B. Kmetiuk, Felipe S. Krawczak, Fernanda P. Machado, Igor A. D. Paploski, Thiago F. Martins, Pedro I. Teider-Junior, Maria C. A. Serpa, Amália R. M. Barbieri, Renato V. W. Bach, Ivan R. Barros-Filho, Leandro C. Lipinski, Andrea P. dos Santos, Marcelo B. Labruna, Alexander W. Biondo

**Affiliations:** 1 Department of Cellular and Molecular Biology, Federal University of Paraná, Curitiba, Paraná, Brazil; 2 Department of Veterinary Medicine, School of Veterinary Medicine and Animal Science, Federal University of Goiás, Goiânia, Goiás, Brazil; 3 Department of Veterinary Science, Federal University of Paraná, Curitiba, Paraná, Brazil; 4 Department of Veterinary Population Medicine, University of Minnesota, St. Paul, Minnesota, United States of America; 5 Department of Preventive Veterinary Medicine and Animal Health, School of Veterinary Medicine and Animal Science, University of São Paulo, São Paulo, Brazil; 6 Department of Medicine, State University of Ponta Grossa, Paraná, Brazil; 7 Assistant professor, Department of Comparative Pathobiology, Purdue University, West Lafayette, Indiana, United States of America; 8 Adjunct professor, Department of Comparative Pathobiology, Purdue University, West Lafayette, Indiana, United States of America; Faculty of Science, Ain Shams University (ASU), EGYPT

## Abstract

**Background:**

*Rickettsia* bacteria are responsible for diseases in humans and animals around the world, however few details are available regarding its ecology and circulation among wild animals and human populations at high transmission risk in Brazil. The aim of this study was to investigate the occurrence of ticks and *Rickettsia* spp. in wild boars, corresponding hunting dogs and hunters.

**Methods:**

Serum samples and ticks were collected from 80 free-range wild boars, 170 hunting dogs and 34 hunters from southern and central-western Brazil, from the Atlantic Forest and Cerrado biomes, respectively, between 2016 and 2018. Serum samples were tested by indirect immunofluorescent-antibody assay (IFA) to detect IgG antibodies against *Rickettsia rickettsii*, *Rickettsia parkeri*, *Rickettsia bellii*, *Rickettsia rhipicephali* and *Rickettsia amblyommatis*. Tick species were identified by morphological taxonomic keys, as previously described. A total of 164 ticks including *A*. *sculptum*, *A*. *brasiliense* and *A*. *aureolatum* were tested in PCR assays for Spotted Fever Group (SFG) *Rickettsia* spp.

**Results:**

A total of 58/80 (72.5%) wild boars, 24/170 (14.1%) hunting dogs and 5/34 (14.7%) hunters were positive (titers ≥ 64) to at least one *Rickettsia* species. A total of 669/1,584 (42.2%) ticks from wild boars were identified as *Amblyomma sculptum*, 910/1,584 (57.4%) as *Amblyomma brasiliense*, 4/1,584(0.24%) larvae of *Amblyomma* spp. and 1/1,584 (0.06%) nymph as *Amblyolmma dubitatum*. All 9 ticks found on hunting dogs were identified as *Amblyomma aureolatum* and all 22 ticks on hunters as *A*. *sculptum*. No tested tick was positive by standard PCR to SFG *Rickettsia* spp.

**Conclusions:**

The present study was the concomitant report of wild boar, hunting dog and hunter exposure to SFG rickettsiae agents, performed in two different Brazilian biomes. Wild boar hunting may increase the risk of human exposure and consequently tick-borne disease Wild boars may be carrying and spreading capybara ticks from their original habitats to other ecosystems. Further studies can be required to explore the ability of wild boars to infecting ticks and be part of transmission cycle of *Rickettsia* spp.

## Author summary

The present study reported serological findings and molecular assays of *Rickettsia* spp and ticks of wild boars, simultaneous to their correspondent hunting dogs and hunters. Seropositivity for *Rickettsia* spp. was higher in wild boars when compared to dogs and humans but was similar between dogs and humans. Despite *Rickettsia* spp. prevalence was statistically higher in southern than central-western Brazil for wild boars, no significance was observed in hunting dogs and hunters. For the first time, *A*. *sculptum* ticks were founded in wild boars from the subtropical climate of southern Brazil. Despite human beings have been considered less exposed to ticks (and therefore rickettsiae) than animals, specific human activities such as wild boar hunting may increase the risk of exposure and consequently tick-borne disease. Wild boars may be carrying and spreading capybara ticks from their original habitats to other ecosystems. These results may provide important findings for public action planning to prevent neglected vector-borne diseases in overlapping areas of wild boars, hunting dogs and hunters. Further studies can be required to explore the ability of wild boars to infecting ticks and be part of transmission cycle of *Rickettsia* spp.

## Introduction

The genus *Rickettsia* (family Rickettsiaceae; order Rickettsiales) comprises gram-negative and obligate intracellular bacteria, which are phylogenetically classified into the spotted fever group (SFG) rickettsiae, the typhus group rickettsiae, the *Rickettsia bellii* group rickettsiae and the *Rickettsia canadensis* group rickettsiae [1]. Tick-borne rickettsioses have been placed into the SFG group, known of causing infection in animals and human beings [2, 3], and participating on enzootic or epizootic cycles among vertebrates and arthropod vectors [4]. Ixodid ticks have been described as the main natural reservoirs and vectors of rickettsiae, with transstadial and transovarial transmission in ticks [5].

*Rickettsia rickettsii*, the main etiological agent of spotted fever in Brazil, has been primarily transmitted to human beings by *Amblyomma sculptum* and *Amblyomma aureolatum* ticks [6, 7, 8]. *Amblyomma sculptum*, characterized by an aggressive behavior and multispecies parasitism, may be the most prevalent tick species in the Cerrado and degraded areas of the Atlantic Rainforest biomes [9, 10]. On the other hand, *A*. *aureolatum* ticks have been mostly found in Atlantic Rainforest fragments, which may provide favorable abiotic conditions and native carnivores as primary hosts [8].

Wild boars (*Sus scrofa*) have been classified by Brazilian laws as exotic invasive species originated by Eurasian wild boars and their hybrids, with nationwide hunting officially permitted (Normative Instruction 03/2013) as a strategy for population control and eradication [11]. Wild boars may invade natural and anthropic areas, not only competing for resources with native wildlife and livestock species, but also sustaining life cycle of ticks and tick-borne diseases [12]. As large-bodied, non-native and the most invasive mammal species, wild boars have been considered as potential hosts of *A*. *sculptum* ticks in Brazilian biomes, particularly the Pantanal floodplains [13, 14].

Hunting dogs (*Canis familiaris*) have been the most popular method for wild boar tracking and hunting in Brazil [15]. Brazilian rural dogs accessing natural areas have been frequently found to show parasitism for *A*. *aureolatum* ticks along with antibodies for *Rickettsia* spp., potentially increasing the risk of human infection when bringing infected ticks to household environment [16, 17, 18].

Density population of capybaras (*Hydrochoerus hydrochaeris*) in spotted fever-endemic areas of southeastern Brazil, mostly related to sugarcane crops production [19], has been 40 times higher than those reported in natural environments [20]. Similarly, wild boar populations have also been associated to several cultivated areas of central-western, southwestern and southern Brazil [21]. Hence, it is reasonable to speculate that the overlapping of wild boar and capybara environments may have a synergic impact on occurrence of ticks and tick-borne diseases.

Despite wild boars, hunting dogs and hunters in Brazil may be exposed to several tick-borne rickettsiae, no study to date has concurrently assessed this potential and alternative life cycle of spotted fever in wild boars, hunting dogs and hunters. Accordingly, the aim of the present work was to determine anti-*Rickettsia* antibodies and presence of ticks in wild boars, hunting dogs and hunters in two different Brazilian biomes (Atlantic Forest and Cerrado).

## Methods

### Study area

**Fig 1:**
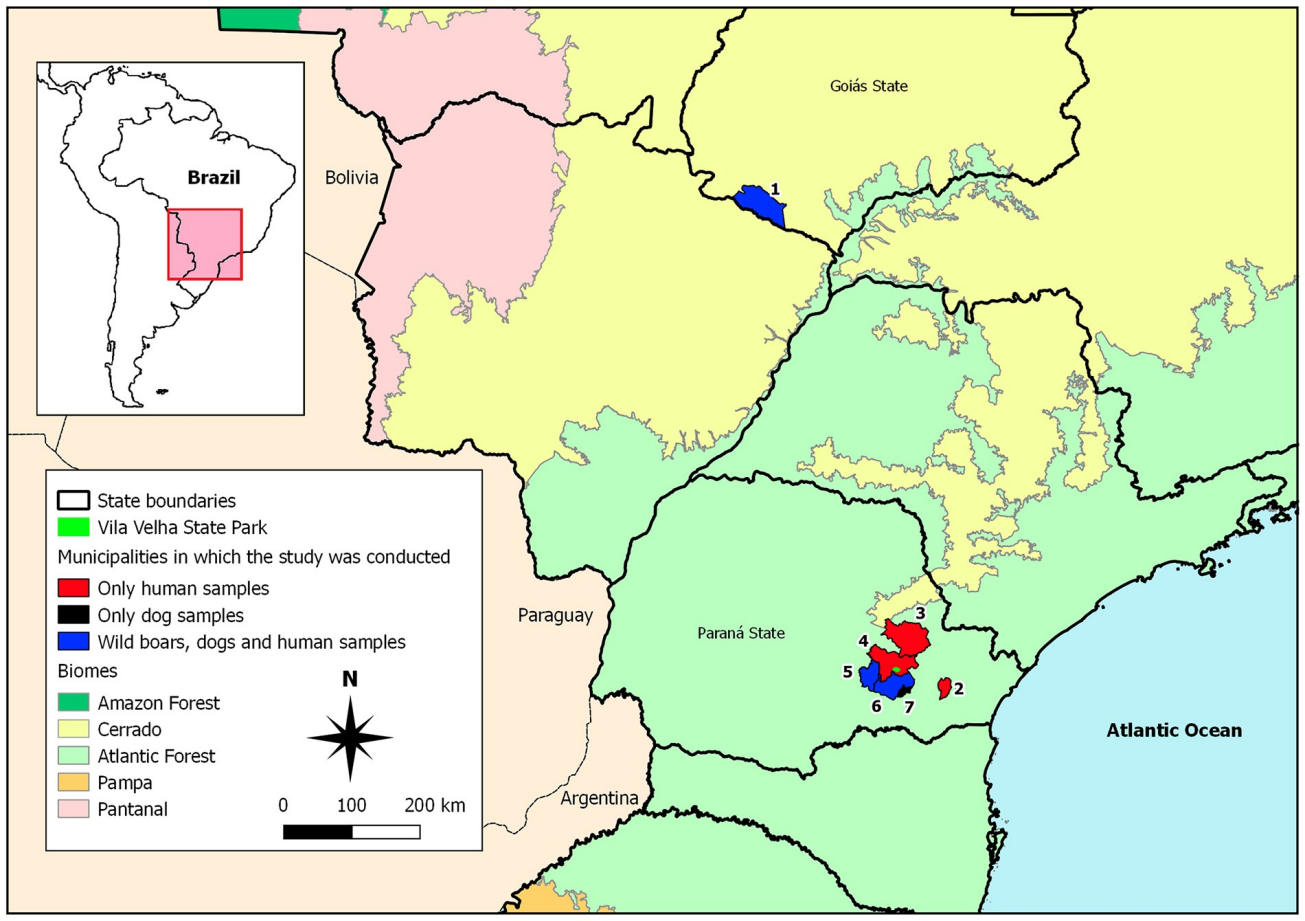
Sampling locations of wild boars, hunting dogs and hunters from southern and central-western Brazil. Locations are numbered as follow: Vila Velha State Park (1), Palmeira (2), Curitiba (3), Castro (4), Ponta Grossa (5), Porto Amazonas (6) and Teixeira Soares (7) from State of Paraná; Aporé (8) from State of Goiás.

This is a descriptive cross-sectional study of boars, hunting dogs, hunters and ticks parasitizing them. The study was conducted in preserved and degraded areas in the Atlantic Forest biome of southern Brazil, including the Vila Velha State Park (belongs to Campos Gerais National Park) and Palmeira, Curitiba, Castro, Ponta Grossa, Porto Amazonas and Teixeira Soares municipalities; and in degraded areas in the Cerrado biome of central-western Brazil, the Aporé (Fig 1).

### Samples and collection

A total of 22 on-field expeditions were carried out from November 2016 to May 2018, which included summer, autumn, winter and spring. Ticks were collected from wild boars, hunting dogs and hunters during all year seasons, which may have covered all possible species and stages. Wild boars blood samples were collected by intracardiac puncture immediately after death, by jugular puncture in dogs and by cephalic puncture in hunters. All samples were collected in tubes without anti-coagulant and kept at room temperature (25 °C) until visible clot retraction, centrifuged at 1,500 revolutions per minute for five minutes, and serum separated and kept at -20 °C until processing.

Wild boars were sampled in agricultural areas of Atlantic Forest and Cerrado biomes following legal hunting laws, along hunting dogs and hunters. Additionally, wild boars at the Vila Velha State Park (belongs to Campos Gerais National Park) were baited, photo-monitored, trapped and euthanized. Both hunting and trapping, along with handling of wild boar samples and ticks were authorized by the Brazilian Environmental Biodiversity System (SISBIO license 61805–2).

Tick sampling of each wild boar was randomly obtained by time-independent collection, with ticks picked on all surfaces of the two body sides to ensure maximum yield. After such hunting activities, resting and blood samplings, dogs were carefully examined for ticks and hunters asked for self-examination for tick presence. All ticks obtained from wild boars, hunting dogs and hunters were collected, preserved in isopropyl alcohol and taken to the laboratory for taxonomic identification, which was performed following standard morphological keys [22, 23, 24]. Hunting dogs underwent annual deworming protocols, along flea and tick control according to visual infestation, done by their owners.

### Laboratory testing of samples

Serum samples were individually tested by indirect immunofluorescent-antibody assay (IFA) for five Brazilian *Rickettsia* isolates: *R*. *rickettsii* strain Taiaçu, *R*. *parkeri* strain At24, *R*. *amblyommatis* strain Ac37, *R*. *rhipicephali* strain HJ5 and *R*. *bellii* strain CL as previously described [25, 26]. Individual sera were initially screened at a 1:64 dilution against each of the rickettsial antigens. A fluorescein isothiocyanate-labeled rabbit anti-pig IgG dilution 1: 1,500 (IgG, Sigma Diagnostics, St. Louis, MO, lot 048K4842) as conjugate was used for hunting wild boars samples, fluorescein isothiocyanate-labeled rabbit anti-dog IgG dilution 1:1,000 (IgG, Sigma Diagnostics, St. Louis, MO, lot 102M4795V) was used as conjugate for the hunting dogs samples, and fluorescein isothiocyanate-labeled rabbit anti-human IgG dilution 1:1,500 (IgG, Sigma Diagnostics, St. Louis, MO, lot 038K4802) as conjugate was used for the hunter samples. In each slide, a serum previously shown to be non-reactive (negative control) and a known reactive serum (positive control) were tested up to the 1:64 dilution. In case of a positive reaction of testing serum, serial dilutions at two-fold increments were tested up to the endpoint titer. Serum showing for a *Rickettsia* species titer at least fourfold higher than those observed for the remaining *Rickettsia* species was considered possibly homologous to the first *Rickettsia* species, as previously determined [25, 26].

A sample of 164 ticks was randomly selected, individually submitted to DNA extraction by the guanidine isothiocyanate technique [27], and individually tested by standard PCR for tick mitochondrial 16S rRNA [28] and rickettsial *gltA* gene [29]. For each PCR run, a negative control (water) and positive control (*Rickettsia vini* DNA) were included [30].

### Ethics

This study has been approved by the Ethics Committee of Animal Use (protocol number 059/2017) of the Federal University of Paraná, officially included as part of the annual activities of the City Secretary of Health at Ponta Grossa and approved by National Human Ethics Research Committee (number 97639017.7.0000.0102). In addition, the in-park trapping and tick collection have been authorized by the Environment Institute of Paraná (authorization number 30/17) and by Chico Mendes Institute of Biology (authorization number 61805–2).

### Data analysis

The absolute and relative frequency of infection was calculated stratifying the observations according to the species and to the region in the country in which samples were collected. The frequency of *Rickettsia* spp. between different species was compared using chi-square test. Observed differences were considered to be significant when the resulting P-value was less than 0.05. A map illustrating the sampling points was constructed using QGIS 2.18.18.

## Results

Blood samples were collected, and ticks searched from 80 wild boars, 170 hunting dogs and 34 hunters. Samples from 60/80 (75.0%) wild boars were obtained by legal hunting (agricultural areas), while 20/80 (25.0%) by trapping (conservation unit area). Among hunting individuals, 24/60 (40.0%) wild boars, 147/170 (86.5%) hunting dogs and 27/34 (79.4%) hunters were sampled at the Atlantic Forest biome, while 36/60 (60.0%) wild boars, 23/170 (13.5%) hunting dogs and 7/34 (20.6%) hunters at the Cerrado biome.

Through serologic analysis for *Rickettsia* spp., 58/80 (72.5%) wild boars, 24/170 (14.1%) hunting dogs, and 5/34 (14,7%) hunters were seropositive for *Rickettsia* spp. (Table 1). In addition, possible antigen involved in a homologous reaction (PAIHR) for *R*. *rickettsii*, *R*. *bellii* or *R*. *rhipicephali* were found in 4/80 (5.0%) wild boars, *R*. *bellii* and *R*. *amblyommatis* in 2/170 (1.17%) hunting dogs (Table 1). Among wild boars, IFA endpoint titers varied from 64 to 1,024 for *R*. *rickettsii* and *R*. *bellii*, 64 to 512 for *R*. *parkeri* and *R*. *rhipicephali*, and 64 to 256 for *R*. *amblyommatis*. IFA endpoint titers in hunting dog samples varied from 64 to 512 for *R*. *rickettsii*, *R*. *bellii*, *R*. *rhipicephali*, *R*. *amblyommatis*, 64 to 256 for *R*. *rickettsii*, and 128 to 1,024 for *R*. *parkeri*. Among hunters, IFA endpoint titers varied from 128 to 256 for *R*. *rickettsii*, 64 to 256 for *R*. *parkeri*, 64 to 128 for *R*. *bellii*, and 64 to 512 for *R*. *rhipicephali* and *R*. *amblyommatis*.

**Table 1:** Results of indirect immunofluorescent-antibody assay (IFA) for five *Rickettsia* species in wild boars, hunting dogs and hunters from southern and central-western Brazil.

Samples	No. tested samples	No. seroreactive individuals to each of the *Rickettsia* species (% seroreactivity)					No. with determined homologous reaction (PAIHR in parentheses)*
		*R*. *rickettsii*	*R*. *parkeri*	*R*. *rhipicephali*	*R*. *amblyommatis*	*R*. *bellii*	
WILD BOARS							
Southern	44	30 (69.8)	22 (51.2)	13 (30.2)	7 (16.8)	23 (53.5)	1 (*R*. *bellii*), 2 (*R*. *rickettsii*)
Central-western	36	9 (24.3)	13 (35.1)	3 (8.1)	0 (0)	10 (27.0)	1 (*R*. *rhipicephali*)
Total	80	39 (48.7)	35 (43.8)	16 (20.0)	7 (8.7)	33 (41.2)	4 (*R*. *bellii*, *R*. *rickettsii*, *R*. *rhipicephali*)
HUNTING DOGS							
Southern	147	5 (3.4)	5 (3.4)	8 (5.4)	11 (7.5)	8 (5.4)	1 (*R*. *bellii*), 1 (*R*. *amblyommatis*)
Central-western	23	0 (0)	0 (0)	0 (0)	0 (0)	3 (13.0)	
Total	170	5 (2.9)	5 (2.9)	8 (4.7)	11 (6.5)	11 (6.5)	2 (*R*. *bellii*, *R*. *amblyommatis*)
HUNTERS							
Southern	27	4 (14.8)	4 (14.8)	4 (14.8)	4 (14.8)	4 (14.8)	
Central-western	7	0 (0)	0 (0)	0 (0)	0 (0)	1 (3.7)	
Total	34	4 (11.8)	4 (11.8)	4 (11.8)	4 (11.8)	5 (18.5)	0

*PAIHR: A homologous reaction was determined when an endpoint titer to a *Rickettsia* species was at least 4-fold higher than those observed for the other *Rickettsia* species. In this case, the *Rickettsia* species involved in the highest endpoint titer was considered the possible antigen involved in a homologous reaction (PAIHR).

Seropositivity for *Rickettsia* spp. was higher in wild boars when compared to dogs (p-value = 0.001) and humans (p-value = 0.001) but was similar between dogs and humans (p-value = 1.000). Despite *Rickettsia* spp. prevalence was statistically higher in southern than central-western Brazil for wild boars (p-value = 0.002), no significance was observed in hunting dogs (p-value = 1.000) and hunters (p-value = 1.000).

Ticks were collected from wild boars, hunting dogs and hunters during all year seasons, covering all possible species and stages. A total of 1,584 ticks were collected from wild boars, including 669 (42.2%) adults of *A*. *sculptum*, 910 (57.4%) *Amblyomma brasiliense* composed by 870 (54.9%) adults and 40 (2.5%) nymphs, 4 (0.24%) larvae of *Amblyomma* spp. and one (0.06%) nymph of *Amblyomma dubitatum*. All 9 ticks founded on hunting dogs were identified as *A*. *aureolatum* adults, and all 22 ticks obtained from the hunters as *A*. *sculptum* nymphs (Table 2). In addition, 24/44 (54.5%) and 8/36 (22.2%) wild boars had an average infestation of 32.7 and 81.5 ticks per animal in southern and central-western Brazil, respectively. *Amblyomma sculptum* was the dominant tick species infesting central-western wild boars, whereas *A*. *brasiliense* was so in southern wild boars. All *A*. *aureolatum*-infested dogs were from the southern region. A total of 164/1,584 (10.4%) ticks, including 162 adults and 2 nymphal, were randomly selected for the detection of SFG rickettsial DNA by PCR. They belonged to one genus including 3 species: 4 *A*. *sculptum* from 2/44 (4.5%) wild boars of southern Brazil, 53 *A*. *sculptum* from 8/36 (22.2%) wild boars of central-western Brazil, 100 *A*. *brasiliense* from 24/44 (54.58%) wild boars of southern Brazil and 7/147 (4.8%) *A*. *aureolatum* from hunting dogs of southern Brazil (Table 2). No rickettsial DNA was detected in these ticks, despite of each of them yielded a visible amplicon in agarose gel through the PCR targeting the tick 16S rRNA gene.

**Table 2:** Species and number of ticks (M: males; F: females; N: nymphs; L: larvae) collected from wild boars, hunting dogs and hunters from southern and central-western Brazil.

Hosts	No. tick sampled/No. animal sampled (%)	No. ticks per species					No. ticks tested by PCR
		*A*. *sculptum*	*A*. *brasiliense*	*A*. *dubitatum*	*Amblyomma* spp.	*A*. *aureolatum*	
WILD BOARS							
Southern	24/44 (54.5)	4 F	638 M, 232 F, 40 N	1 N	4 L	-	4 *A*. *sculptum*; 100 *A*. *brasiliense*
Central-western	8/36 (22.2)	447 M, 218 F	-	-	-	-	31 *A*. *sculptum*
Total	32/80 (40.0)	669	910	1	4	-	35 *A*. *sculptum*; 100 *A*. *brasiliense*
HUNTING DOGS		-	-	-	-	-	
Southern	7/147 (4.8)	-	-	-	-	1 M, 8 F	7 *A*. *aureolatum*
Central-western	0/23 (0)	-	-	-	-	-	
Total	7/170 (4.2)	-	-	-	-	9	7 *A*. *aureolatum*
HUNTERS		-	-	-	-	-	
Southern	0/27 (0)	-	-	-	-	-	
Central-western	7/7 (100)	19 M, 3 F	-	-	-	-	22 *A*. *sculptum*
Total	7/34 (20.58)	22	-	-	-	-	22 *A*. *sculptum*

## Discussion

The present study reports serological findings and molecular assays of *Rickettsia* spp and ticks of wild boars, simultaneous to their correspondent hunting dogs and hunters. Seropositivity for *Rickettsia* spp. was higher in wild boars when compared to dogs and humans but was similar between dogs and humans. Despite results have apparently shown a higher seropositivity of hunting dogs and hunters in southern than in central-western Brazil, differences were not statistically significant probably due to a reduced statistical power between the prevalence of groups formed by stratification according to region. Since this was not the aim of the present study, further studies should be conducted to fully establish differences on serological rickettsial titers of wild boars, hunting dogs and hunters among different Brazilian regions.

The difference of seropositivity between southern and central-western wild boars could be related to dominant tick species, namely *A*. *sculptum* in central-western and *A*. *brasiliense* in southern Brazilian regions. Serological results herein may indicate that, if the *A*. *sculptum* populations infesting wild boars, dogs and hunters in central-western Brazil were infected by any SFG pathogenic rickettsiae, the infection rate would be very low or only few populations would be infected. In fact, the low rickettsial seropositivity in central-western wild boars, hunting dogs and hunters could be a result of the rare rickettsial infection in *A*. *sculptum* ticks [31, 32]. The only exceptions may be the spotted fever endemic areas of southeastern Brazil, where some populations of this tick species may be infected by *R*. *rickettsii* [33, 34]. Thus, all *A*. *sculptum* tested were negative for *Rickettsia* spp. in molecular analyses.

On the other hand, the much higher seropositivity of wild boars, hunting dogs and even hunters in southern Brazil may suggest that the *A*. *brasiliense* populations from this region would be infected by one or more SFG rickettsiae, yet to be identified in further studies. To the best of our knowledge, no rickettsial agent has been identified in *A*. *brasiliense* yet.

Wild boars have been suggested to play a potential role in the eco-epidemiology of rickettsioses. In Catalonia, Spain, 12/23 (52.2%) and 19/23 (82.6%) wild boars sampled were seropositive to *Rickettsia slovaca*, classified into the SFG and associated with *Dermacentor marginatus* ticks [35]. In Mississippi, USA 17/58 (29,3%) feral swine were seropositive to the SFG pathogen *R*. *parkeri* [36]. Although capybaras have long been recognized as the major host of *A*. *sculptum* and amplifier species for *R*. *rickettsii* infection in Brazil [37], future studies should be conducted to fully establish the role of wild boars as hosts, amplifiers and their association to human cases of *R*. *rickettsii*-caused spotted fever.

Despite human beings have been considered less exposed to ticks (and therefore rickettsiae) than animals [38], specific human activities such as hunting may increase the risk of exposure and consequently of disease. Not surprisingly, individuals from rural areas who visit forest areas, rivers and waterfalls have also shown higher incidence of spotted fever infection [39]. Unfortunately, no information was found about hunting habits of a non-fatal human case of spotted fever illness notified in a nearby area of southern Brazil and other two cases notified in nearby area of central-western Brazil [40, 41], which hinders the risk assessment for this activity in regard to *Rickettsia* spp. transmission.

Important to remark that, as mentioned before, hunting is currently unlawful in Brazil. Actually, wild boar hunting has been officially considered as “controlling non-protected invasive exotic species”, therefore the only legal regulated form of hunting activity to date in Brazil (Normative Instruction 03/2013) [11].

The tick species obtained herein on wild boars have been previously involved in *Rickettsia* spp. transmission to dogs and human beings [10, 33]. Association of hunting practices with seroreactivity to *Rickettsia* spp. has been attributed to a higher exposure to *Amblyomma* spp. while hunting, since these ticks have been primarily associated with wildlife in Brazil [42]. Further studies that better estimate the prevalence of infection in these populations are required to better design control strategies.

Although restricted to Brazilian Pantanal biome (floodplains), feral pigs, *Sus scrofa* L. (Artiodactyla, Suidae), have been previously suggested as hosts to *A*. *sculptum* [13, 14]. For the first time, *A*. *sculptum* ticks were found in two wild boars of subtropical climate from southern Brazil. In a recent study about the distribution of *A*. *sculptum* in Brazil, it was shown that this tick is absent from most of the southern region, possibly due to more severe winter temperatures [10]. The repeatedly findings herein of both engorged adults (successfully fed) and engorged nymphs (different stages) of *A*. *brasiliense* and *A*. *sculptum* on wild boars in the Atlantic Forest and Cerrado biomes, respectively (Table 2), have shown host adaptation and spreading to two more Brazilian biomes, suggest that these tick species might be adapting and spreading to areas previously thought as unsuitable for their survival.

All ticks collected from wild boars at the conservation unit area of Atlantic Forest were identified as *A*. *brasiliense*, probably due to predominant high humidity and lower temperatures, important for this tick species development [43], naturally maintained in such areas by native peccaries (*Tayassu* spp.) as primary hosts. However, the relative higher presence (tick average per animal) of adult and nymph stages in wild boars may suggest overlapping of ecological niche, and higher traveling body area as competent *A*. *brasiliense* hosts. Although *A*. *brasiliense* has been considered aggressive to human beings [44, 45] and such scenario may impact on higher tick and tick-borne disease spreading, *R*. *rickettsii* transmission by *A*. *brasiliense* ticks have been observed only under experimental conditions [46], and absent in molecular surveys on natural environments [17, 47, 48, 49]. Not surprisingly, no *A*. *brasiliense* tested herein by standard PCR was positive to *Rickettsia* spp.

Hunting dogs in the present study were only found with *A*. *aureolatum*, corroborating to previous studies in dogs from rural areas nearby rainforest fragments and hunter activities [50, 51, 52]. In a previous study in southern Brazil, 19/133 (14.3%) rural dogs were reported with ticks, including *A*. *aureolatum* [53]. These ticks were the second most prevalent among rural dogs of another study from southern Brazil, representing 52/153 (33,9%) of the collected ticks [54]. *Amblyomma aureolatum* ticks have shown high susceptibility to *R*. *rickettsii* infection, and dogs as one of the most important hosts in spotted fever-endemic areas [51]. The infection by *R*. *rickettsii* may contribute to lower survival and reproduction in *A*. *aureolatum* females, resulting in low infection rates (<10%) under natural conditions [7]. While this assumption could be associated to the absence of rickettsial DNA in the *A*. *aureolatum* ticks of the present study, we are aware that we have tested only a small sample of ticks, precluding a more rational conclusion. Seven hunters in the present study became infested by *A*. *sculptum* ticks after hunting. *Amblyomma sculptum* is the most frequent human-biting tick in Brazil, and also one of the main vectors of *R*. *rickettsii* in the country [10, 33]. These findings highlight hunters as a potential risk group for tick-borne spotted fever in Brazil.

Since *Rickettsia* spp usually infect and remain inside host endothelial cells, molecular detection has usually failed when investigating blood samples [55]. Under experimental *R*. *rickettsii*-infected tick infestation, rickettsial DNA has been detected by PCR in only one of 32 (3.1%) blood samples of infected capybaras, despite serological titers up to 16,384 [56]. In the same study, despite serological titers up to 32,768, direct intraperitoneal inoculation has failed to provide rickettsial DNA detection in blood samples. Thus, in the present study, no molecular investigation was made on blood samples of wild boars, hunting dogs and hunters.

Wild boars may be carrying and spreading capybara ticks from their original habitats to other ecosystems. In Florida, USA, wild boars have been found over long distances and different ecosystem, increasing contact to multiple tick species in their preferential microhabitat [12]. Besides higher-energy requirements obtained in long distance incursions, adult wild pigs have also larger body area [57] than capybaras, which might be an important characteristic of wild boars in spreading ticks in Brazil. Altogether, such overlapping distribution of wild boars and capybaras in Brazil may lead to synergistic spreading of vector ticks, particularly of *R*. *rickettsii*-caused spotted fever, locally called as Brazilian spotted fever.

Wild boars may post an additional treat due to their highly adaptative capacity, spreading themselves to both intact and degraded areas of all six Brazilian biomes, including Atlantic Forest (rainforest), Cerrado (tropical savanna), Pampas (open fields), Pantanal (flood plains), Amazon (rainforest) and Caatinga (semi-arid), as recently recognized by the Brazilian Ministry of Agriculture (map in S1 Fig) [58]. As already mentioned, Brazilian Spotted fever and other rickettsial agents have reportedly overlapped capybara occurrence, therefore wild boars may carry ticks and tick-borne diseases outside capybara original areas, currently restricted to gallery forests and seasonally flooded savannas such as the Atlantic Forest, Pantanal and Cerrado [59]. In such scenario, authors hypothesize that wild boars may overspread ticks and rickettsial diseases to Brazilian biomes lacking capybaras as the Caatinga biome, a dry area found on northern, northeastern and southeastern Brazil.

In addition, the Brazilian Ministry of Environment has warned about the ineffectiveness of wild boar natural population control by Brazilian native predators, mostly due to low populations of already critically endangered species as pumas (*Puma concolor*) and jaguars (*Panthera onca*), associated to wild boar groups weighting up to 220 kg, defending themselves by sticking together and returning the attacks with potential wounds by bites and tusks [60]. Although hunting increase may be necessary to successfully control wild boar populations, authors suggest a governmental nationwide establishment of sanitary hunting guidelines, conducted always with tick-bite prevention and early recognition of rickettsial disease symptoms.

The present study has shown seropositivity for at least one *Rickettsia* species in wild boars, hunting dogs and hunters. Despite an expected lower exposure of humans to ticks (and therefore rickettsiae) than animals, specific human activities such as wild boar hunting may increase the risk of human exposure and consequently tick-borne disease. Wild boars may be carrying and spreading capybara ticks from their original habitats to other ecosystems lacking capybaras, with no effective natural predators. These results may provide important findings for public health action to prevent vector-borne diseases in overlapping areas of capybaras, wild boars, hunting dogs and hunters. Further studies should be conducted to fully establish the wild boar ability to infect ticks and its role on *Rickettsia* spp. transmission cycle.

## Supporting information

S1 Fig Perception of wild boar presence throughout Brazilian cities, according to official livestock inspectors, Ministry of Agriculture and Livestock, Brazil (www.agricultura.gov.br). (TIF)
