# Supplementary figures and images for: Ticks and serosurvey of anti-Rickettsia spp. antibodies in wild boars (Sus scrofa), hunting dogs and hunters of Brazil
Source: PLoS Negl Trop Dis. 2019 May 30;13(5):e0007405. doi: 10.1371/journal.pntd.0007405 (PMC6542515; doi:10.1371/journal.pntd.0007405)

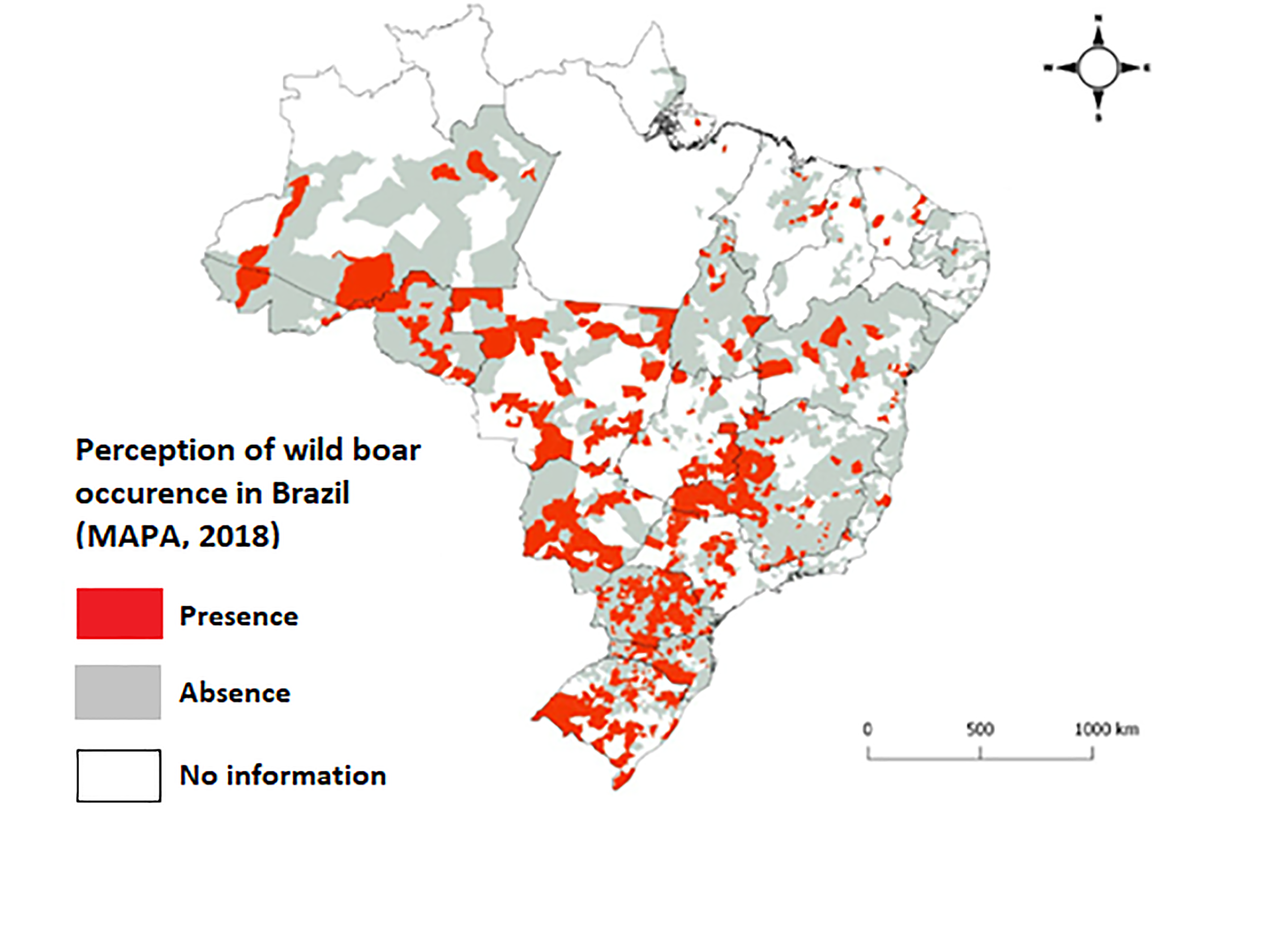

Supplement: S1 Fig — (TIF) [file pntd.0007405.s001.tif]
